# Serum cathepsin K levels of patients with longstanding rheumatoid arthritis: correlation with radiological destruction

**DOI:** 10.1186/ar1461

**Published:** 2004-11-10

**Authors:** Martin Skoumal, Günther Haberhauer, Gernot Kolarz, Gerhard Hawa, Wolfgang Woloszczuk, Anton Klingler

**Affiliations:** 1 Institut für Rheumatologie der Kurstadt Baden in Kooperation mit der Donauuniversität Krems, Austria; 2 Rheumasonderkrankenanstalt der SVA der gewerblichen Wirtschaft, Baden, Austria; 3 Biomedica Medizinprodukte GmbH & CO KG, Vienna, Austria; 4 L Boltzmann Institut für experimentelle Endokrinologie, Vienna, Austria; 5 Theoretical Surgery Unit, Department of General and Transplant Surgery, University Hospital, Innsbruck, Austria

**Keywords:** bone remodelling, cathepsin K, osteoclast activation, rheumatoid arthritis

## Abstract

Cathepsin K is a cysteine protease that plays an essential role in osteoclast function and in the degradation of protein components of the bone matrix by cleaving proteins such as collagen type I, collagen type II and osteonectin. Cathepsin K therefore plays a role in bone remodelling and resorption in diseases such as osteoporosis, osteolytic bone metastasis and rheumatoid arthritis. We examined cathepsin K in the serum of 100 patients with active longstanding rheumatoid arthritis. We found increased levels of cathepsin K compared with a healthy control group and found a significant correlation with radiological destruction, measured by the Larsen score. Inhibition of cathepsin K may therefore be a new target for preventing bone erosion and joint destruction in rheumatoid arthritis. However, further studies have to be performed to prove that cathepsin K is a valuable parameter for bone metabolism in patients with early rheumatoid arthritis.

## Introduction

Progressive bone and cartilage destruction in arthritic joints leads to irreversible joint destruction, and subsequently to functional declines and work disability [1,2]. New biomarkers such as cartilage oligomeric matrix protein [3,4], osteoprotegerin [5-7] or receptor activator of NF-κB ligand [8-10] have been developed to describe the local bone and cartilage process in affected joints.

Cathepsin K is a cysteine protease that plays an essential role in osteoclast function and in the degradation of protein components of the bone matrix. It is produced by bone resorbing macrophages and synovial fibroblasts, and it cleaves proteins such as collagen type I, collagen type II and osteonectin [11]. Cathepsin K therefore plays a role in bone remodelling and resorption in diseases such as osteoporosis, osteolytic bone metastasis and rheumatoid arthritis (RA) [12,13].

Cathepsin K is a tissue-specific protease associated with pycnodysostosis, a rare genetic disorder that manifests itself in bone abnormalities such as short stature, acroosteolysis of distal phalanges and skull deformities [14,15]. Cathepsin K knockout mice develop an osteopetrosis. Inhibition of cathepsin K may therefore prevent bone resorption, as could be demonstrated in bone metastasis from breast cancer [16]. Osteoprotegerin has been shown to inhibit the expression of cathepsin K, the main enzyme involved in bone resorption.

The aim of this study was to measure serum levels of cathepsin K in RA and to prove that cathepsin K is a parameter of bone remodelling and resorption in a nonselected cohort of patients with longstanding RA. This patient group shows a variation of age, inflammatory level and Larsen score. We divided this cohort into different groups, according to age, inflammatory level, disease-modifying antirheumatic drug (DMARD) therapy, radiological progression and disease activity, to verify cathepsin K as an age-independent and laboratory inflammatory parameter-independent protease.

## Materials and methods

Serum levels of cathepsin K were measured in the sera of 100 patients suffering from RA according to the criteria of the American Rheumatism Association [17]. Clinical and laboratory data are presented in Tables 1 and 2. The control group consisted of nonselected healthy blood donors from a central blood bank (*n *= 50; 21 female, 29 male) aged 18–65 years.

Most of the patients received DMARDs. The most frequently used DMARD was methotrexate, followed by leflunomide, sulfasalzopyrine and gold. Furthermore, azathioprine and chloroquine but no biological therapy were prescribed (Table 3).

Each examination consisted of a full interview, the assessment of functional disability and a standardised physical examination, which included a joint examination for tenderness (Ritchie score), pain on motion, soft tissue swelling, 44-swollen joint count and swollen proximal interphalangeal score [18,19].

The disease activity of RA was measured by the disease activity score (≤ 2.4, low activity; > 2.5 and ≤ 3.7, mean activity; > 3.7, high activity). The radiological progression in RA was calculated by the Larsen score [20].

The blood examination at each visit consisted of the determination of cathepsin K, the erythrocyte sedimentation rate, the haemoglobin level, the thrombocyte count, the serum rheumatoid factor (RapiTex^® ^RF; Dade Behring, Liederbach, Germany), antinuclear antibodies (indirect immunfluorescent technique, ANA Fluor Kit 240^®^; Diasorin, Stillwater, MN, USA) and C-reactive protein (CRP) (Rheumajet CRP^®^; Biokit, Barcelona, Spain).

The variables of age, sex, duration of disease, visual analogue scale of general health and morning stiffness, treatment with DMARDs and reason for their discontinuation, and the Steinbrocker stage [21] were also recorded. Serum was obtained in the morning from the routinely taken blood samples and was centrifuged immediately. The samples were kept at -80°C prior to determination of cathepsin K. The serum used for the measurement of cathepsin K was the remainder from routinely drawn blood examinations on the day of hospitalisation; no examination was performed only for quantification of cathepsin K. Clinical data were used from a database developed for the long-term observation of patients with RA in our clinic.

An enzyme immunoassay for cathepsin K developed by Biomedica Austria (Vienna, Austria) was used. The Cathepsin K test kit is an enzyme immunoassay designed to determine cathepsin K directly in biological fluids (serum, plasma, cell culture supernatants). The ELISA used in this study is based on antibodies specific for amino acids 1–20 and amino acids 196–210 of the mature enzyme. The antibodies were produced by immunisation of sheep with peptides of that amino acid sequence coupled to Keyhole Limpet Hämocyanine (primary immunisation, 0.5 mg; boost, 0.25 mg). Antisera were purified using the biotinylated peptides coupled to streptavidine sepharose (Amersham-Pharmacia Biotech Ltd, Little Chalfont, UK). A synthetic cathepsin K (Pichem GmbH, Graz, Austria) was used as the calibrator. Signal generation was accomplished by labelling with horseradish peroxidase.

Briefly, the assay procedure consisted of incubating 50 μl sample with 200 μl horseradish peroxidase-labelled detection antibody on capture antibody precoated plates overnight at room temperature. After a washing step to remove unbound detection antibody, tetramethyl benzidine was added as the substrate. The reaction was stopped after 30 min by adding 50 μl of 0.9% H_2_SO_4_. The yellow colour that is directly proportional to the amount of cathepsin K present in the sample was measured on a standard microplate reader at 450 nm with 620 nm as the reference. The detection limit of the assay was calculated as 1.1 pmol/l (0 standard + 5 × standard deviation).

No cross-reactivity to cathepsin E, cathepsin D, cathepsin B and cathepsin L or rheumatoid factors was detected.

Statistical methods included Spearman correlation analysis, the Wilcoxon two-sample test the Kruskal–Wallis test and analysis of variance, if appropriate. *P *< 5% was considered statistically significant.

## Results

The cathepsin K serum levels of the patients with RA (median first–third quartile range, 54.8 pmol/l) compared with the healthy control group (median first–third quartile range, 12.7 pmol/l) were significantly elevated (*P *= 0.0003) (Table 4).

The Larsen score ranged from 0 to 164 (median score, 39). The Spearman rank correlation showed a statistically significant correlation between cathepsin K and the Larsen score (*P *= 0.004). The highest levels of cathepsin K were observed in patients with the highest Larsen scores. We divided the cohort into three Larsen groups with equal numbers of patients (Larsen score < 18 points, Larsen score between 19 and 74 points, and Larsen score ≥ 75 points). Cathepsin K levels showed an increase with the augmentation of radiological destruction (*P *= 0.035) (Table 5).

Cathepsin K seems to be independent or only weakly correlated with laboratory inflammation parameters. It was not associated with CRP (*P *= 0.27), but weak correlations were found with the erythrocyte sedimentation rate (*P *= 0.03) and the disease activity score of the whole cohort (*P *= 0.04). However, the division of the disease activity score into three groups with low activity, medium activity and high activity did not show any difference. We could not find any correlation with sex and age (whole group/division into two patient groups ≤ 65 years and ≥ 66 years, *P *= 0.32), whereby the two groups were comparable in disease activity (3.53 versus 3.12), laboratory parameters (CRP, 25.4 mg/l versus 25.9 mg/l), clinical score (Ritchie score, 14 versus 9) and radiological score (Larsen score, 47 versus 62).

The most frequently used DMARD was methotrexate (*n *= 42), followed by leflunomide (*n *= 10) and sulfasalzine (*n *= 10). Twenty-two patients had no DMARD at the time of examination (Table 3). The lowest cathepsin K levels were evident in the leflunomide group, but no significant difference between these groups could be demonstrated.

## Discussion

Bone resorption and formation is a well-balanced system and is mediated by osteoclasts. Cathepsin K is essential for bone resorption, which depends on the production of cathepsin K by osteoclasts and its secretion into the extracellular department. This leads to a degradation of the organic matrix between the osteoclasts and the bone surface [22]. *In vivo *the activation of cathepsin K occurs intracellularly, before secretion into the resorbing lacunae and the onset of bone resorption, whereby local factors may regulate the processing of procathepsin K to mature cathepsin K [23]. In accordance with this, synovial fibroblasts are also involved in joint destruction and in the pathogenesis of RA. Hou and colleagues found that cathepsin K has a potent aggrecan-degrading activity, whereby the aggrecan cleavage products increase the collagenolytic effects of this protease on collagen type I and type II. They were able to show that cathepsin K is also a critical protease in cartilage degradation by synovial fibroblasts [24]. Increased expression of cathepsin K around lymphocytic infiltrates in synovial tissue seems to facilitate the movement of mononuclear cells through the perivascular matrix [25]

Proinflammatory cytokines such as IL-1β and tumour necrosis factor alpha influence the expression of cathepsin K. Its overexpression in the rheumatoid synovium, induced by IL-1β and tumour necrosis factor alpha due to the increase of cathepsin K-expressing cells, proves this protease to be a valuable tool for bone research, and cathepsin K also may become a new and highly specific biomarker for RA [26].

Votta and colleagues demonstrated high levels of cathepsin K expression in osteoclasts at sites of extensive bone loss. According to this, they developed a peptide aldehyde inhibitor of cathepsin K that inhibits osteoclast-mediated bone resorption in foetal rat long bone organ cultures and even in a human osteoclast-mediated assay *in vitro*. This inhibitor leads to a significantly reduced bone loss [27]. Furthermore, structure activity studies on a series of reversible ketoamide-based inhibitors of cathepsin K have led to the identification of potent and selective inhibitors [28].

Wittrant and colleagues demonstrated osteoprotegerin to be an inhibitor of cathepsin K. Osteoprotegerin is an osteoblast-secreted decoy receptor that inhibits osteoclast differentiation and activation. Human osteoprotegerin inhibits cathepsin K and tartrate-resistant acid phosphatase, both osteoclast markers, but stimulates the expression of tissue inhibitor of metalloproteinases-1 [29]. These results are a further step in the development of new therapies for the prevention of bone destruction.

In the synovium of RA, the cathepsin K protein was localised in synovial fibroblasts, stromal multinucleated giant cells and CD68^+ ^macrophage-like synoviocytes. Highly interesting is the expression of cathepsin K by fibroblasts and giant cells at sites of cartilage erosions. This was two to five times higher compared with osteoarthritic synovium. In normal synovium, cathepsin K expression was not increased and was restricted to fibroblast like cells [26,30-32]. The overexpression of cathepsin K in RA synovia proves that this protease is responsible for the degradation of articular tissue in rheumatoid joints and in normal synovial tissue.

To our knowledge, no study has previously investigated the serum levels of cathepsin K in RA. Our results demonstrate that cathepsin K is elevated in the serum of patients with RA compared with that of a healthy control group (Table 4). The upregulation of cathepsin K and the correlation with the Larsen score as a parameter for radiological changes (Table 5) mirrors the destruction of bone structures in inflammatory diseases like RA. The measurement of cathepsin K seems an inexpensive tool that is independent of CRP and shows only a weak correlation with the erythrocyte sedimentation rate.

Further studies should investigate whether elevated cathepsin K levels precede osseous destruction or whether they occur as result of them. In the first case, determination of cathepsin K could be an important additional tool to decide on aggressive forms of disease-modifying antirheumatic therapies.

## Conclusion

This is the first study that demonstrates increased cathepsin K levels in the serum of patients with RA. As could be shown in the synovia of RA, the elevated serum levels of this protease are significantly correlated with the joint destruction, which in this study was assessed by the Larsen score. Cathepsin K seems to be a valuable parameter for the assessment of bone metabolism in patients with established RA and its measurement will probably contribute to developing targeted therapies for the prevention of further bone destruction. However, more studies need to be performed to verify the presence of cathepsin K in patients with early RA and its value as a prognostic factor for bone destruction in RA

## Abbreviations

CRP = C-reactive protein; DMARD = disease-modifying antirheumatic drug; ELISA = enzyme-linked immunosorbent assay; IL = interleukin; NF = nuclear factor; RA = rheumatoid arthritis.

## Competing interests

Dr G Hawa and Prof. W Woloszczuk are members of BIOMEDICA who developed the Cathepinsin K kit, but they did not receive any financial benefits.

## Authors' contributions

MS is the corresponding author, and GH and GK are coauthors of the manuscript. GH and WW developed the cathepsin K ELISA kit. AK performed the statistical analysis.

## Figures and Tables

**Table 1:** Clinical parameters of 100 rheumatoid arthritis (RA) patients

	Disease duration (years)	Age at manifestation (years)	Age (years)	Morning stiffness (min)	Ritchie score	Larsen score	44 swollen joint count	Disease activity score
Mean	11.7	52.0	62.9	31.9	11.3	54.8	7.4	3.3
Minimum	0.5	18.0	20.0	0.0	0.0	0.0	0.0	0.4
Maximum	56.0	75.0	83.0	130.0	42.0	164.0	28.0	6.0
Standard deviation	11.6	12.4	11.0	37.8	10.3	49.5	6.8	1.4
Median	8.0	53.0	63.0	15.0	10.0	38.0	6.0	3.6

**Table 2:** Laboratory parameters of 100 rheumatoid arthritis (RA) patients

	Rheumatoid factor (U/l)	Erythrocyte sedimentation rate (mm/hour)	C-reactive protein (mg/l)	Leucocytes (g/l)	Cathepsin K (pmol/l)
Mean	298.8	30.3	25.0	7.2	304.7
Standard deviation	1142.3	20.9	23.4	2.2	681.0
Median	27.0	30.0	20.0	7.0	54.8

**Table 3:** Distribution of disease-modifying antirheumatic drug in 100 rheumatoid arthritis patients

	Disease-modifying antirheumatic drug						
	
	None	Methotrexate	Leflunomide	Sulfasalazopyrine	Gold	Chloroquine	Others
Number	22	42	10	10	6	4	6

**Table 4:** Correlations of cathepsin K with clinical, laboratory and radiological parameters

	Mean	Standard deviation	*n*	Coeffficient	Probability > |*r*|
Variable 1					
Cathepsin K	304.66	677.607			
Variable 2					
Age (years)	62.89	11.1581	100	0.0543	0.5915
Rheumatoid factor (U/l)	298.818	1136.49	100	0.4761	< 0.0001
Morning stiffness (min)	32	38.7233	100	0.1320	0.1905
Erythrocyte sedimentation rate (mm/hour)	30.27	20.8404	100	0.2200	0.0279
C-reactive protein (mg/l)	24.96	23.5032	100	0.1121	0.2670
Ritchie score	11.31	10.3394	100	0.1353	0.1797
Proximal interphalangeal score	1.51	2.69865	100	0.2560	0.0101
Disease activity score	3.33283	1.43345	100	0.2093	0.0376
Larsen score	54.77	49.3335	100	0.2856	0.0040

**Table 5:** Increase of cathepsin K levels with the augmentation of the Larsen score

Larsen group		Larsen score			Cathepsin K			Kruskal–Wallis test
		
	*n*	Minimum	Median	Maximum	Minimum	Median	Maximum	
< 18	32	0.0	7.5	17.0	0.0	26.5	3352.0	
≥ 19 and < 74	34	18.0	38.0	67.0	0.0	70.9	1721.6	
≥ 75	34	75.0	105.5	164.0	0.0	88.8	3453.2	
Total	100	0.0	39.0	164.0	0.0	54.8	3453.2	*P *= 0.035
